# Impact of Mannitol Bladder Distension in the Intraoperative Detection of Ureteral Kinking During Pelvic Floor Surgery

**DOI:** 10.1007/s00192-024-05745-z

**Published:** 2024-02-23

**Authors:** Marta Barba, Alice Cola, Clarissa Costa, Matteo Frigerio

**Affiliations:** 1 Fondazione IRCCS San Gerardo Dei Tintori, Monza, Italy; 2 Milano-Bicocca University, Via G.B. Pergolesi 33, 20900 Monza, Italy

**Keywords:** Complications, Cystoscopy, Mannitol, Pelvic organ prolapse, Vaginal hysterectomy, Uterosacral ligament suspension

## Abstract

**Introduction and Hypothesis:**

Ureteral injuries are the most feared complications of gynecological surgery and therefore intraoperative recognition is of the utmost importance. Intraoperative cystoscopy represents the diagnostics of choice to investigate ureteral patency thanks to the direct visualization of ureteral flows after administration of infusion mediums. In this study, we aimed to compare the diagnostic performance of saline versus mannitol intraoperative cystoscopy in terms of false negatives in a large cohort of patients.

**Methods:**

We retrospectively analyzed data of patients who underwent vaginal hysterectomy and high uterosacral ligament suspension for POP. Patients were divided in two groups based on the use of saline or mannitol medium for intraoperative cystoscopy. Postoperative daily control of serum creatinine was performed until discharge, as well as urinary tract imaging, in symptomatic patients.

**Results:**

A total of 925 patients underwent vaginal hysterectomy followed by high USL suspension for POP. Saline and mannitol medium were used in 545 patients and 380 patients respectively. Postoperative ureteral injuries were identified in 12 patients, specifically in 2% of the saline group and in 0.3% of the mannitol group.

**Conclusions:**

The use of mannitol instead of saline as a bladder distension medium was able to significantly reduce the occurrence of postoperative ureteral sequelae.

## Introduction

Ureteral injuries are likely to be the most feared complication of gynecological surgery, and are estimated to occur in up to 10.9% of procedures [1]. Intraoperative recognition is of the utmost importance, in order to avoid diagnostic delay, which results in prolonged hospitalization, additional surgical or endoscopic procedures, and potentially serious sequelae including sepsis, urinoma or urinary fistula formation, and nonfunctioning kidney [2]. Moreover, ureteral complications involve expenses and the risk of medicolegal actions. Intraoperative detection of ureteral injury offers the opportunity for timely proper management during the index surgery, which may involve suture removal, stent positioning, or neoureterocystostomy [3]. This allows a decrease in serious sequelae, costs, and litigations.

The close relationship of the ureters to the uterus, vagina, and infundibulopelvic vessels puts them at risk at the time of hysterectomy. Moreover, surgery performed for prolapse frequently may involve placing additional sutures in proximity to the ureters and near the site of entry of the ureters into the bladder. This might be particularly relevant for prolapse native tissue repair, which returning to prominence owing to lower costs and a lack of mesh-related complications [4, 5]. These involve apical support restoration through the use of ligamentous or muscular structures [6–9]. Among all procedures, uterosacral ligament suspension is particularly versatile and effective [10–15], but it is also associated with the highest risk of ureteral injury, ranging between 1.8% and 10.9% of procedures [1, 16].

**Fig. 1:**
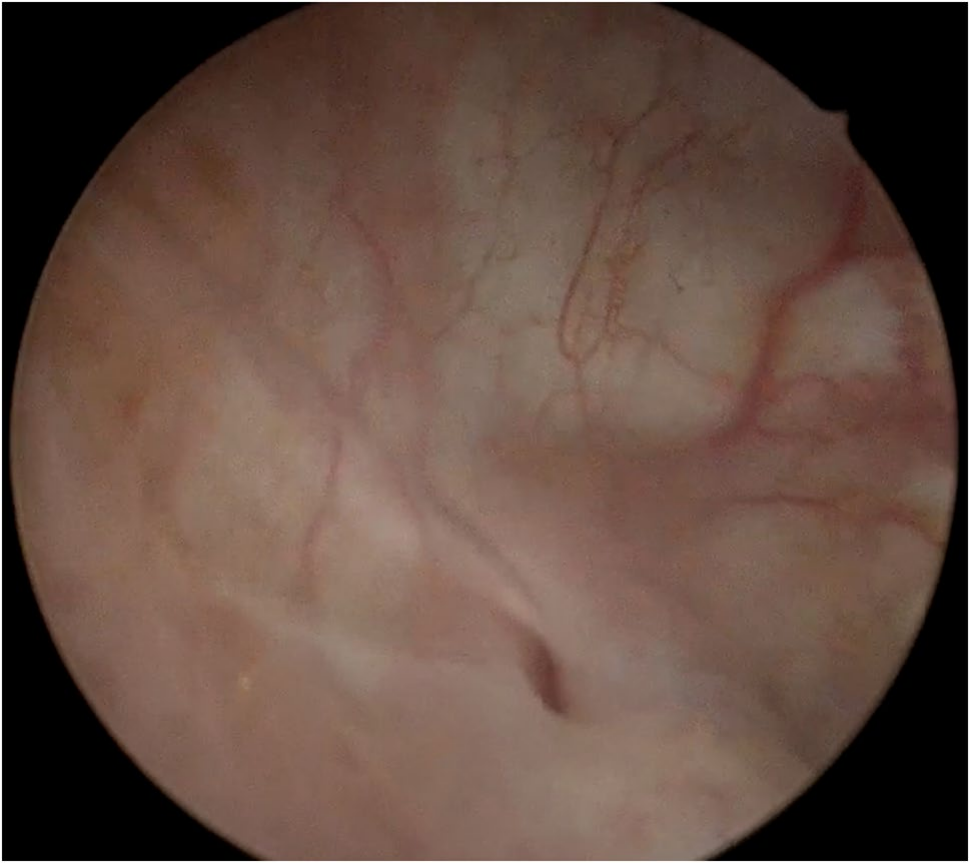
Mannitol solution bladder distension: evidence of an enhanced ureteral jet is provided

Intraoperative cystoscopy represents the diagnostics of choice to investigate ureteral patency [17]. This was usually assessed through direct visualization of dyed ureteral jets after indigo carmine endovenous administration. In recent years, indigo carmine was no longer obtainable, owing to shortage and then discontinuation [18]. In the absence of indigo carmine, ureteral jets have less visible results, affecting the diagnostic performance of cystoscopy in defining ureteral patency. Moreover, the visualization of the movement of the ureteral meatus in the absence of a clear jet is unreliable and may possibly afford the surgeons a false sense of reassurance. In this post-indigo carmine era, pelvic floor surgeons performing intraoperative cystoscopy have not been left with a clear alternative in evaluating ureteral patency. Standard saline solution as a bladder-distending medium became the main option thanks to its low cost and wide distribution, but recently, the use of mannitol solution has been proposed to allow better visualization of the flow of urine during cystoscopy, without any intravenous administration of dyes [19]. The rationale is to take advantage of the difference in viscosity between mannitol and the urine spilling from the ureteral orifice (Fig. 1). Despite being promising in terms of surgeon satisfaction, ease of use, visualization of the ureteral jets, and adverse events in a cohort of 32 patients, to date no data about the impact of mannitol on detecting ureteral injuries have to our knowledge been published [19].

With this study, we aimed to compare the diagnostic performance of saline versus mannitol intraoperative cystoscopy after high-risk surgical procedures, evaluating postoperative ureteral sequelae rates as a marker of diagnostic false negatives in a large cohort of patients.

## Materials and Methods

The data of patients who underwent vaginal hysterectomy followed by high uterosacral ligament suspension for POP between January 2013 and December 2022 were retrospectively analyzed. Inclusion criteria for surgery were symptomatic pelvic organ prolapse, prolapse greater than stage II according to pelvic organ prolapse quantification (POP-Q), and/or failure of conservative techniques in women in menopause or without the desire to reproduce. Exclusion criteria for surgery were mainly anesthesiological contraindications. We divided the population into two groups based on the use of either saline intraoperative cystoscopy (group 1 January 2013 to December 2017) or mannitol medium intraoperative cystoscopy (group 2 January 2018 to December 2022).

All surgical procedures were performed by experienced pelvic floor surgeons. In the given period, this technique represented our standard procedure for primary uterovaginal prolapse repair in the case of menopausal women or fertile-age women not requiring uterine preservation. Patients underwent vaginal hysterectomy and salpingectomy, whereas bilateral oophorectomy was performed, if technically feasible, according to menopausal status, age, oncological risk, and the patient’s will after proper counseling. After the hysterectomy, small bowels were packed out of the operative field with a long gauze, and the Breisky–Navratil retractor was properly positioned to expose USLs. Uterosacral ligaments were then bilaterally transfixed in their intermediate portion (at the level of or above the ischial spine plane), with 2 or 3 double-needle monofilament long-term absorbable 0 sutures (Assufil monofilamento; Assut Europe, Rome, Italy). In order to reduce the risk of ureteral entrapment, USLs were bilaterally transfixed from ventral to dorsal. USL sutures were then passed both anteriorly and posteriorly through the peritoneum, the apex of the vaginal fascia, and the vaginal mucosa. When anterior repair was performed, vesicovaginal fascia plication was carried out before fascia transfixion with USL sutures. USL sutures were tightened to close both the peritoneum and the vaginal cuff. Cul-de-sac obliteration was not performed routinely but only in specific cases (e.g., enterocele). If necessary, the posterior repair was performed after the sutures were tightened.

Diagnostic cystoscopy was performed at the end of the surgical time to assess ureteral bilateral patency. In group 1 intraoperative cystoscopy was performed with saline medium distension. In group 2, diagnostic cystoscopy was performed using mannitol solution as bladder distension medium. In the case of a lack of ureteral jet visualization, sutures were removed and possibly repositioned during the index surgery, and cystoscopy was repeated. On the contrary, in the case of the impression of the ureteral jets, the ureters were considered not obstructed and surgery was completed. During the postoperative course, a daily serum creatinine check was performed until discharge, and in the case of an increase in creatinine or lumbar back pain, urinary tract imaging was requested. Urine analyses were performed after surgery only in the case of urinary symptoms.

During the 1-month follow-up visit, once again the presence of lumbar-back pain was investigated, and eventually, kidney imaging was performed. Any ureteral complication that required conservative or surgical management was noted and considered a false negative of intraoperative cystoscopy.

The study was approved by the institutional review board of Fondazione IRCCS San Gerardo dei Tintori in Monza, Italy. Data were entered into the database by one author and double-checked by one other author. Statistical analysis was performed using JMP software version 9.0 (SAS Campus Drive, Cary, NC, USA). Data are reported as mean (SD). Differences were tested using Fisher's test for noncontinuous data. A *p* value < 0.05 was considered statistically significant.

## Results

The retrospective chart review identified 925 patients who underwent vaginal hysterectomy followed by high USL suspension for POP between January 2013 and December 2022. For all patients, operative, postoperative, and 1-month follow-up data were available. Based on the period and the use of either basal or mannitol solution-augmented cystoscopy, 545 patients were assigned to group 1 (basal cystoscopy, January 2013 to December 2017), whereas 380 were included in group 2 (mannitol solution, January 2018 to December 2022). Patients' characteristics are summarized in Table 1. Specifically, there were no differences between groups in terms of patients' age, parity, body mass index (BMI), preoperative POP-Q, and anterior repair.

**Table 1:** Population characteristics: comparison between group 1 (saline cystoscopy) and group 2 (mannitol cystoscopy)

	Group 1 (*n* = 545)	Group 2 (*n* = 380)	*p* value
Age	64.0 ± 10.4	65.3 ± 9.9	0.074
Parity	2.2 ± 1.1	2.2 ± 1.0	0.929
Body mass index	25.1 ± 3.7	25.0 ± 3.7	0.630
Aa	1.1 ± 1.5	1.2 ± 1.5	0.087
Ba	1.3 ± 1.6	1.5 ± 1.6	0.076
C	0.4 ± 2.7	0.5 ± 3.0	0.444
gh	3.7 ± 0.6	3.8 ± 0.6	0.193
pb	2.8 ± 0.5	2.8 ± 0.5	0.840
tvl	10.0 ± 1.2	10.1 ± 1.2	0.282
Ba	−1.4 ± 1.3	−1.3 ± 1.4	0.266
Bp	−1.4 ± 1.3	−1.2 ± 1.5	0.171
D	−4.0 ± 2.6	−3.7 ± 2.8	0.066
Anterior repair	459 (84.2%)	336 (88.4%)	0.071

Postoperative ureteral injuries were identified in 12 patients (1.3%) during the whole period. Specifically, postoperative ureteral injuries occurred in 11 patients in group 1 (2.0%), and in 1 woman in group 2 (0.3%). This corresponded to a significant increase in the intraoperative detection of ureteral injuries after the introduction of mannitol solution bladder distension (*p* = 0.019) by observing a lack of ureteral jets during cystoscopy. In these cases, intraoperative removal of uterosacral ligament sutures restored ureteral jets without any further complications. Details of all ureteral injuries are provided in Table 2. In 5 out of 12 cases, the lesion was identified during the index hospital stay, whereas in the remaining 7 cases, readmission after discharge was necessary. In 2 patients conservative treatment with cystoscopy ureteral stenting was feasible and effective in solving the kinking. However, in most cases (10 out of 12; 83.3%) surgical treatment was necessary, usually through transvaginal suture removal; in 1 case ureteroneocystostomy was required to manage a persistent ureterovaginal fistula. After ureteral injury resolution, no sequelae were observed, except for partial loss of function of the kidney in the patient with the longest surgery-to-treatment interval (38 days).

**Table 2:** Details of all postoperative ureteral injuries

*N*°	Group	Clinical/instrumental findings	Management/resolution	Outcomes
#1	1	Readmission for right lumbar pain on 9th POD	US positioning failure	Resolution
		Serum creatinine increase and right hydroureteronephrosis	TV sutures (apical) removal and US positioning	No sequelae
#2	1	Readmission for right lumbar pain on 18th POD	US positioning failure	Resolution
		Serum creatinine increase and right hydroureteronephrosis	TV sutures (apical and anterior repair) removal	No sequelae
#3	1	Readmission for left pyelonephritis in 15th POD	US positioning failure	Resolution
		Serum creatinine increase and left hydroureteronephrosis	TV sutures (apical and anterior repair) removal	No sequelae
#4	1	Acute renal failure in 1st POD and right hydroureteronephrosis	US positioning failure	Resolution
			TV sutures (apical and anterior repair) removal	No sequelae
#5	1	Readmission for left uretero-vaginal fistula (enuretic urinary incontinence at 1-month visit)	US positioning but fistula persistence;	Resolution; No sequelae
			Open Leadbetter–Politano left ureteroneocystostomy after 3 months	(No ureteral stricture nor vesico-ureteral reflux)
#6	1	Readmission for abdominal pain on the 11th POD	US positioning failure	Resolution
		Serum creatinine increase and left hydroureteronephrosis	TV sutures (apical) removal and left US positioning	No sequelae
#7	1	Anuria and acute renal failure in 1st POD	US positioning failure	Resolution
		Bilateral hydroureteronephrosis	TV sutures (apical and anterior repair) removal	No sequelae
#8	1	Right lumbar pain on the 3rd POD	US positioning failure	Resolution
		Serum creatinine increase and right hydroureteronephrosis	TV sutures (apical) removal	No sequelae
#9	1	Readmission for right lumbar pain in 6th POD	Nephrostomy plus US positioning	Resolution
		Right hydroureteronephrosis and right ureterovaginal fistula	TV suture (apical and anterior repair) removal	No sequelae
#10	1	Right lumbar pain on the 1st POD	US positioning	Resolution
		Serum creatinine increase and right hydroureteronephrosis		No sequelae
#11	1	Readmission for left pyelonephritis on the 38th POD	US positioning	Resolution
		Serum creatinine increase and left hydroureteronephrosis		Left hypofunctional kidney at renal scintigraphy
#12	2	Bilateral lumbar pain in 2nd POD;	TV suture removal (apical and anterior repair) and bilateral US positioning	No sequelae
		Mild serum creatinine increase (1.3 mg/dl) and bilateral hydroureteronephrosis		

*POD* postoperative day, *TV* transvaginal, *US* ureteral stent

Apart from the type of intraoperative cystoscopy performed, no other risk factors for postoperative ureteral injuries were identified, including patients’ characteristics (age, parity, and BMI), preoperative POP-Q, and anterior repair (Table 3).

**Table 3:** Risk factor analysis: comparison between patients with unrecognized ureteral injuries and controls

	Ureteral injuries (*n* = 12)	Controls (*n* = 913)	*p* value
Age	66.3 ± 6.7	64.5 ± 10.3	0.774
Parity	2.5 ± 1.2	2.2 ± 1.1	0.285
Body mass index	23.4 ± 2.5	25.1 ± 3.7	0.132
Aa	1.2 ± 2.0	1.1 ± 1.5	0.911
Ba	1.5 ± 1.9	1.4 ± 1.6	0.793
C	1.2 ± 2.9	0.4 ± 2.9	0.545
gh	3.7 ± 0.7	3.8 ± 0.6	0.854
pb	2.8 ± 0.3	2.8 ± 0.5	0.830
tvl	10.0 ± 1.5	10.0 ± 1.2	0.773
Ba	−1.1 ± 1.7	−1.3 ± 1.4	0.873
Bp	−1.1 ± 1.7	−1.3 ± 1.4	0.894
D	−3.6 ± 2.2	−3.9 ± 2.7	0.768
Anterior repair	11 (91.7%)	784 (85.9%)	0.567

## Discussion

Although ureteral injuries occur infrequently after benign gynecological procedures, they are more common with pelvic floor operations, which clearly predispose to lower urinary tract injuries. Specifically, ureteral kinking is considered the main pitfall of USL suspension during prolapse repair. Its recognition during the procedure is of the utmost importance to avoid long-term sequelae. However, the opportunity to perform universal cystoscopy is under debate owing to costs, invasiveness, and lack of training [2]. In addition, routine cystoscopy is characterized by false negatives as it can miss ureteral injuries caused by delayed thermal effect, post-ligature swelling, and incomplete defects. Last, the unavailability of indigo carmine has left pelvic floor surgeons with uncertainty about the diagnostic performance of saline cystoscopy and possible alternatives.

Our study demonstrated in a cohort of 925 patients that the use of mannitol instead of saline as a bladder distension medium was able to significantly reduce the occurrence of postoperative ureteral sequelae (*p* = 0.019). The only false-negative cystoscopy occurring in the mannitol group was likely related to a partial ureteral obstruction that cystoscopy was unable to detect, despite visualization of the ureteral jets, as demonstrated by the minimal increase in serum creatinine level (1.3 mg/dl). In all cases, ureteral injuries identified in the postoperative setting corresponded to Clavien–Dindo grade III complications, as they required either surgical or endoscopic intervention. In most cases, management did not involve long-term sequelae. However, timing is likely to be a crucial factor, as the only long-term sequelae (partial loss of kidney function) occurred in the patient with the longest surgery-to-complication recognition interval (38 days).

The importance of relying on accurate diagnostics is even more critical considering that the occurrence of unrecognized ureteral injury looks like an unpredictable event, as demonstrated by the lack of identifiable risk factors.

Although mannitol bladder distension has been shown to be promising in terms of surgeon satisfaction, ease of use, visualization of the ureteral jets, and adverse events, to date there have been no data on the impact of mannitol on detecting ureteral injuries [19].

The rationale for taking advantage of the difference in viscosity between the bladder medium and the urine spilling from the ureteral orifice has been previously evaluated for other mediums such as dextrose solution [18]. Like mannitol, dextrose solution does not rely on colors to show ureteral efflux. Its difference in viscosity allows a clearer contrast with urine, providing effective visualization. Furthermore, as this medium does not require renal excretion and is directly administered in the bladder, it does not cause excessive extension of operative time [20]. Several studies showed that dextrose instillation seems to be slightly more effective at showing ureteral efflux than other media such as oral phenazopyridine (probability of success 0.99 vs 0.92) and sodium fluorescein IV (probability of success 0.99 vs 0.97) [21]. Despite these advantages, it is considered that the instillation of dextrose solution may slightly increase the risk of urinary tract infections by creating a favorable environment for bacteria. Several studies investigated this notion by comparing dextrose with other solutions. Results showed no apparent significant differences in UTI symptoms after adjusting risk factors such as BMI, diabetes, age, and day of catheter removal [22]. In addition, the risk of hyperglycemia in diabetic patients seems to be low as the medium is placed for a short amount of time in the bladder, determining negligible systemic side effects and absorption [23]. Despite these results, postoperative UTIs are still considered a side effect of dextrose cystoscopy in high-risk patients and therefore it is not considered the first option in cystoscopy procedures.

To the best of our knowledge, this is the first study comparing postoperative outcomes in terms of ureteral sequelae of mannitol versus saline bladder distension intraoperative cystoscopy in high-risk gynecological procedures. Another strength is the large cohort of patients, which allowed differences to be identified, even in rare events such as unrecognized ureteral injuries. One limitation is represented by the retrospective design of the study. Another limitation may be found in the unbalanced numerosity of the two groups. However, the decrease in the number of surgical procedures performed in the second 5-year period was an effect of the reduction in elective activity during the COVID-19 pandemic period [24].

Our study demonstrated that the use of mannitol instead of saline as the bladder distension medium was able to significantly improve ureteral jet visualization and therefore increase the diagnostic performance of the ureteral patency test by decreasing false negatives, thus reducing undiagnosed ureteral injuries that could require postoperative interventions.
